# Misleading Results in Posttraumatic Stress Disorder Predictive Models Using Electronic Health Record Data: Algorithm Validation Study

**DOI:** 10.2196/63352

**Published:** 2025-08-27

**Authors:** Thomas M Crow, Eric Lin, Kelly L Harper, Michael L Crowe, Terence M Keane, Brian P Marx

**Affiliations:** 1 Behavioral Science Division, National Center for Posttraumatic Stress Disorder, VA Boston Healthcare System, 150 S Huntington Ave, 13B, Boston, MA, 02130, United States, 1 6172329500; 2 Massachusetts Veterans Epidemiology Research and Information Center (MAVERIC), VA Boston Healthcare System, Boston, MA, United States; 3 McLean Institute for Technology in Psychiatry, McLean Hospital, Belmont, MA, United States; 4 Department of Psychiatry, Boston University Chobanian & Avedisian School of Medicine, Boston, MA, United States

**Keywords:** clinical prediction models, posttraumatic stress disorder, PTSD, electronic health records, machine learning, posttraumatic, stress disorder, semistructured interview, veterans, clinic, clinics, sensitivity analyses, clinical informatics, misleading information, mental health, misleading result

## Abstract

**Background:**

Electronic health record (EHR) data are increasingly used in predictive models of posttraumatic stress disorder (PTSD), but it is unknown how multivariable prediction of an EHR-based diagnosis might differ from prediction of a more rigorous diagnostic criterion. This distinction is important because EHR data are subject to multiple biases, including diagnostic misclassification and differential health care use resulting from factors such as illness severity.

**Objective:**

This study aims to compare predictive models using the same predictors to predict an EHR-based versus semistructured interview-based PTSD diagnostic criterion, quantify model performance discrepancies, and examine potential mechanisms that account for performance differences.

**Methods:**

We compared the performance of several machine learning models predicting EHR-based PTSD diagnosis to models predicting semistructured interview-based diagnosis in a nationwide sample of 1343 US veterans who completed Structured Clinical Interview for *DSM-5* (*Diagnostic and Statistical Manual of Mental Disorders, Fifth Edition*) (SCID-5) interviews and had clinic visit data extracted from the Veterans Affairs (VA) EHR. We developed 2 sets of predictive models using 3 algorithms (elastic net regression, random forest, and XGBoost), with a nested cross-validation scheme consisting of an initial train-test split and 10-fold cross-validation within the training set for each type of model. All models used a nearly identical set of predictors including 29 EHR-based visit count variables and 8 demographic variables.

**Results:**

Diagnostic concordance between EHR-based PTSD diagnosis and SCID-5-based PTSD diagnosis was 73.3%, with 17.8% false negatives and 8.9% false positives for EHR-based diagnosis. Models predicting EHR-based PTSD performed very well (area under the receiver operating characteristic curve [AUC] .85-.9; Matthews correlation coefficient [MCC] .58-.69), whereas those predicting interview-based PTSD performed only moderately well overall (AUC .71-.76; MCC .24-.28). Sensitivity analyses showed that participants’ frequency of VA visits played a role in these differences, such that the density of EHR data (proportion of nonzero visit counts across EHR variables) was more associated with EHR-based PTSD diagnosis (b=−0.18, SE 0.02, *P*<.001) than with SCID-5 interview–based PTSD diagnosis (b=−0.06, SE 0.01, *P*<.001).

**Conclusions:**

Predictive models of PTSD built using only EHR data demonstrated inflated performance metrics relative to models predicting diagnosis from a rigorous structured clinical interview. This performance discrepancy appears driven by circular relationships between health care use patterns and EHR-based diagnosis that do not affect external diagnostic criteria. Researchers building clinical prediction models should not assume that diagnosis in the EHR is a sufficient proxy for the true criterion of interest. Clinicians and researchers should be cautious in interpreting clinical prediction models using only EHR data, as their real-world utility may be less than performance metrics suggest.

## Introduction

Mental health researchers have increasingly turned to large datasets from electronic health record (EHR) systems in hopes of gaining new insights into mental disorders. In posttraumatic stress disorder (PTSD) research, multiple studies have relied solely or primarily on EHR diagnostic data to characterize the prevalence and correlates of PTSD in different subpopulations [1-4] or to identify PTSD based on existing EHR data [5-8]. In the context of the Veterans Health Administration (VHA) system, where data on PTSD are available and prevalent, using machine learning or similar statistical techniques to identify PTSD among veterans holds the promise of automating screening efforts, decreasing administrative burden, and identifying veterans whose mental health struggles might otherwise be missed in routine care.

Despite this promise, biases in EHR data may limit the conclusions that they facilitate [9,10]. In psychiatric research, one source of bias lies in diagnostic misclassification [11]. Research with veterans enrolled in VHA has indeed demonstrated that a considerable number of veterans are misclassified with respect to PTSD diagnostic status, in both directions (ie, false positives and false negatives) [12-14]. Other biases associated with EHR data arise from how patients interact with health care systems. For example, patients with more health problems are more likely to frequently visit hospitals or clinics and therefore have fewer missing data; poorer health is thus one of several variables identified in past research that can result in biases that threaten models’ external validity [9,15,16].

Even as PTSD research increasingly relies on EHR-based PTSD diagnostic codes, it is not known how differently predictive analytic models might perform when using EHR data to predict PTSD status in the EHR versus PTSD status independent of the EHR, as determined by well-trained diagnosticians using a well-validated diagnostic interview. The aforementioned diagnostic discrepancies and differential patterns of service use are among the sources of bias in EHR data that may distort the results of predictive models.

In this study, we demonstrate the impact on predictive performance when using EHR-based features to predict (1) veteran PTSD status in the VHA EHR versus (2) veteran PTSD status external to the EHR, as determined by trained assessors using a standardized, psychometrically strong diagnostic interview. We used data from the Project VALOR (Veterans After-Discharge Longitudinal Registry) registry of veterans across the United States to assess the performance of multiple machine learning algorithms predicting independent, standardized clinical interview-diagnosed PTSD from 37 variables comprising visit-related EHR data and several demographic variables. We contrasted the performance of these diagnostic interview-based PTSD models with nearly identical models predicting the presence of PTSD diagnosis in the EHR, highlighting differences in predictive performance when the outcome is a high-quality criterion external to the EHR (ie, diagnostic interview) versus when both the outcome and the features (besides demographics) are internal to the EHR. In this exploratory analysis, the comparison of models predicting interview-based PTSD with those predicting EHR-based PTSD elucidates potential differences in predictive accuracy and underscores the importance of using good diagnostic measures in model development.

## Methods

### Participants

Participants were the subset of 1343 veterans from across the United States who completed the clinical interview in wave 2 of Project VALOR, a national registry of Army and Marine Corps veterans deployed in service of Operation Enduring Freedom (OEF) or Operation Iraqi Freedom (OIF). In Project VALOR, women veterans were oversampled at a 1:1 ratio, and veterans were sampled at a 3:1 ratio for “probable PTSD,” defined by history of at least 2 instances of a PTSD diagnosis by a mental health provider in the EHR. For inclusion in VALOR, veterans must have had at least one mental health evaluation at a VA facility. See Rosen et al [17] for detailed recruitment procedures. There and in this paper, we report how we determined our sample size, all data exclusions (if any), all manipulations, and all measures in the study.

### Measures

#### Demographic Variables

Age, gender, sexual orientation, and race variables from the Project VALOR study data are summarized in Table 1, broken down by interview-based PTSD status. The demographic variables chosen are those typically readily available in the VA EHR, making them plausible candidates for a clinical prediction model using only EHR data. Participants could indicate multiple racial categories using checkboxes. Participants identified as 79% White (n=1054), 17% Black (n=229), 3.4% Native American (n=45), 2.2% Asian (n=29), and 12% Hispanic/Latino (n=166). The category “Native Hawaiian or other Pacific Islander” was dropped in the near-zero-variance modeling step because less than 1% of participants (n=9) checked this box.

**Table 1.** Demographics. For the age variable, *P* values are given for the Wilcoxon rank-sum test; for categorical variables, Pearson chi-squared test was used when all expected cell counts were greater than 5; otherwise, Fisher exact test was used. Table constructed with the gtsummary R package [18].

Characteristics	Overall (N=1343)	SCID-5^a^ PTSD–^b^ (n=360)	SCID-5 PTSD+^c^ (n=983)	*P* value
Age (years)				.05
Mean (SD)	40.65 (9.80)	39.94 (9.92)	40.91 (9.75)	
Median (IQR)	38 (32-48)	37 (32-47)	38 (32-48)	
Missing	3	2	1	
Gender, n (%)				.08
Female	687 (51)	170 (47)	517 (53)	
Male	656 (49)	190 (53)	466 (47)	
Race and ethnicity, n (%)				
Hispanic/Latino	166 (12)	38 (11)	128 (13)	.21
Missing	9	1	8	
Native American, n (%)	45 (3.4)	8 (2.2)	37 (3.8)	.16
Missing	1	0	1	
Asian, n (%)	29 (2.2)	7 (1.9)	22 (2.2)	.74
Missing	1	0	1	
Black, n (%)	229 (17)	37 (10)	192 (20)	<.001
Missing	1	0	1	
White, n (%)	1054 (79)	310 (86)	744 (76)	<.001
Missing	1	0	1	
Sexual orientation, n (%)				.98
Homosexual	53 (4)	14 (3.9)	39 (4)	
Heterosexual	1233 (92)	333 (93)	900 (92)	
Bisexual	34 (2.5)	8 (2.2)	26 (2.7)	
Other or I Don’t Know	17 (1.3)	4 (1.1)	13 (1.3)	
Missing	6	1	5	

a SCID-5: Structured Clinical Interview for *DSM-5* (*Diagnostic and Statistical Manual of Mental Disorders, Fifth Edition*)

b PTSD–: posttraumatic stress disorder negative.

c PTSD+: posttraumatic stress disorder positive.

#### PTSD Module of the Structured Clinical Interview for *DSM-5* (*Diagnostic and Statistical Manual of Mental Disorders, Fifth Edition*) (SCID-5)

The Structured Clinical Interview for *DSM-5* (*Diagnostic and Statistical Manual of Mental Disorders, Fifth Edition*) (SCID-5) is a comprehensive semistructured diagnostic interview assessing *DSM-5* psychiatric disorders [19]. In this study, the SCID-5 was administered by telephone by trained, doctoral-level assessors who attended regular reliability meetings. As the primary outcome, this study used a dichotomous PTSD diagnosis present either currently (at wave 2 interview) or since wave 1. Reliability of the SCID-5 PTSD module was excellent (κ=.82) for a randomly selected subsample of 100 VALOR participants.

#### EHR Data

For all participants, EHR data were extracted from the VA centralized health data repository. The EHR variables were chosen on the basis of their clinical relevance for veterans in the VA system. This represents a secondary use of these EHR variables, which were chosen and gathered for a separate study and recoded for this study. These data comprised 3 sources: (1) 20 variables representing counts of visits with outpatient mental health (OPMH) codes at VA (eg, psychotherapy, medication management, and psychological testing); (2) 2 variables representing counts of visits in the emergency department or urgent care (ED/UC) clinics coded with any psychiatric diagnosis or a PTSD diagnosis specifically; and (3) 7 variables representing counts of pharmacy dispensations of drugs across several categories (eg, opioid, benzodiazepines, and antidepressants). Throughout this paper, we refer to pharmacy “visits” rather than “dispensations” for consistency with the other EHR variables, although pharmacy variables technically represent drug dispensations. All EHR variables were expressed as counts, from zero (no visits or dispensations observed) to infinity. The 2 PTSD-relevant variables comprised counts of visits (from OPMH or ED/UC) where any PTSD diagnostic code was assigned (*ICD-9* [*International Classification of Diseases, Ninth Revision*] code 309.81 or *ICD-10* [*International Statistical Classification of Diseases and Related Health Problems 10th Revision*] codes F43.10–F43.12). Dichotomization of PTSD variables for use as dependent variables is described below in “Statistical Analysis.” A full list of EHR variables can be found in Multimedia Appendix 1.

The EHR data spanned 2131 days total, up to 1950 days within-participant, and always before each participant’s Project VALOR wave 2 PTSD assessment. In this timeframe, most but not all participants (89.4%, n=1200) had at least one observed visit in a VA clinic or pharmacy; 143 participants had no observed visits, and thus all EHR variables were coded as zero. The median number of unique visit dates observed per participant was 116 (range 0‐838).

### Statistical Analysis

All analyses were conducted in R version 4.3 (R Core Team) [20], using tidymodels [21] packages. Near-zero variance EHR features (ie, predictors) were removed before modeling. Categorical demographic variables were dummy coded, and all predictors were standardized before modeling. We trained 2 sets of 3 algorithms. Each set shared nearly identical features but differed with respect to the outcome variable, which were: (1) PTSD diagnosis as assessed with the SCID-5 (“SCID-PTSD” models) and (2) any history of documented PTSD diagnosis in the EHR within the timeframe described previously in the “EHR Data” section (“EHR-PTSD” models).

PTSD was coded dichotomously, with 0 for “No.” The SCID-PTSD criterion was coded 1 for positive PTSD diagnosis based on the SCID-5 (prevalence=73.2%). The EHR-PTSD criterion was coded 1 if either of the 2 EHR-based PTSD count variables (described previously in the “EHR Data” section) indicated that a participant had a PTSD diagnosis assigned at one or more visits (prevalence=64.3%). This definition was based on the goal of discovering any evidence of a PTSD diagnosis and on the concern that using a higher cutoff could amplify confounding between the number of visits and the presence of diagnosis. Because other studies have used 2 or more diagnoses as the PTSD criterion, we also examined model performance when the cutoff was 2+ PTSD diagnoses as a sensitivity analysis. Performance metrics for these models are in Multimedia Appendix 1.

Both sets of models comprised 3 classification algorithms: (1) elastic net (regularized logistic) regression using the glmnet package [22], (2) random forest using the ranger package [23], and (3) gradient boosting using the XGBoost package [24]. For each algorithm, multiple hyperparameters were tuned with a grid search. See Multimedia Appendix 1 for hyperparameter tuning information and optimal hyperparameter combinations. Very small amounts of missing demographics data (<2%) were handled with the recipes package [25] using *k*-nearest neighbor imputation, a method that performs well with mixed data types [26]. For both outcomes, calibration for the best-performing algorithm—that is, agreement between observed and model-predicted probabilities—was assessed with logistic calibration curves and integrated calibration index [27].

For all models, we used a nested cross-validation (CV) strategy: first, we took an initial holdout (test) set of one-fifth of the data (n=270). In the remaining training set (n=1073), we conducted 10-fold CV (repeated 10x) for each model, using area under the receiver operating characteristic curve (AUC) to select the optimal hyperparameter combination for each model. Results focus on the predictive performance of these models in the holdout (test) data. In addition to AUC, we present several other performance metrics for the final models for transparency about multiple aspects of performance. These include sensitivity, specificity, negative predictive value (NPV), positive predictive value (PPV), and the Matthews correlation coefficient (MCC). Also known as the phi coefficient, the MCC (range −1 to 1) is a comprehensive measure that addresses several limitations of other metrics [28] and can be interpreted similarly to a Pearson correlation coefficient *r* (in fact, the Pearson correlation coefficient calculated for 2 binary variables will equal the MCC).

In post hoc analyses, we examined the possibility that EHR data sparsity—the proportion of zeroes across a participant’s EHR-based variables—might be related to differences in performance between models using EHR-based versus interview-based outcomes. We reasoned that the shared data source between the EHR-based PTSD outcome and the EHR-based predictors may have subjected these data to shared bias-generating mechanisms that may be partly reflected in variability in the density of participants’ EHR data. In contrast, the interview-based PTSD outcome should not be subject to these mechanisms as much or at all, because the SCID-5 data were gathered independently of EHR data or health care use after initial sampling for Project VALOR. To this end, we used (1) Welch *t* tests, (2) multiple regression analysis with data sparsity as the outcome, and (3) we reran all machine learning models for both outcomes, importance-weighted by data sparsity such that participants with more densely populated EHR data were given more importance in model fitting. The analyses were not preregistered. Further, for purposes of transparency, our Multimedia Appendix 1 contains additional information about packages and functions used, final model hyperparameters, and so on.

### Ethical Considerations

Study procedures were approved by the Institutional Review Board at the VA Boston Healthcare System and the Human Research Protection Office of the U.S. Army Medical Research and Materiel Command (Protocol 2739: “Project VALOR: Trajectories of Change in PTSD in Combat-Exposed Veterans”). Informed consent, including a description of study risks and benefits, was obtained from all study participants. Participants provided consent for researchers to access and retrieve data from their VA medical records throughout the study and received $100 (USD) for the completion of the questionnaire and assessment.

## Results

### Overview

**Figure 1.**
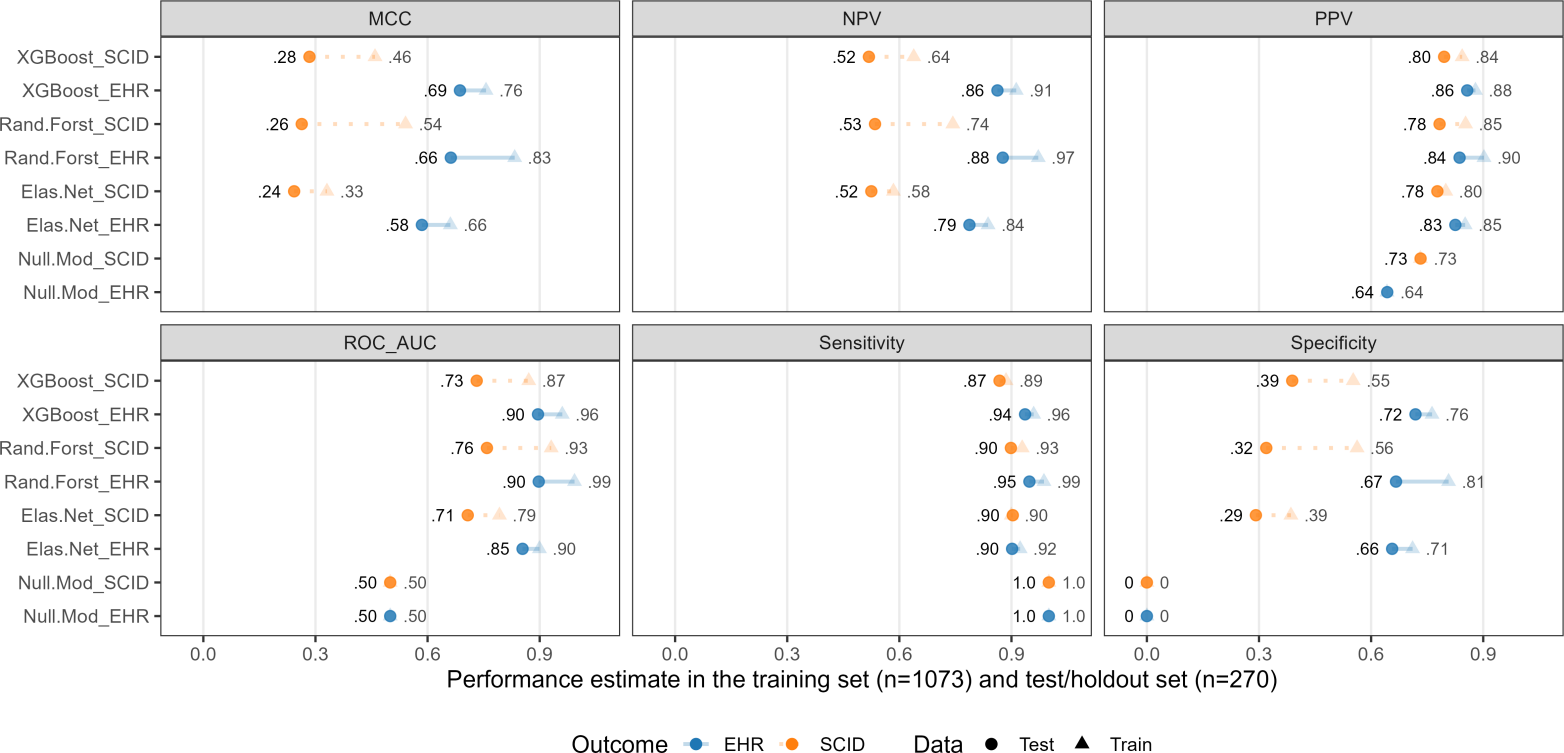
Performance metrics for primary SCID- and EHR-PTSD models. SCID corresponds to models whose outcome was PTSD (yes/no) based on the SCID-5 semistructured diagnostic interview. EHR corresponds to models whose outcome was PTSD (yes/no) based on any history of diagnosis in the Veterans Affairs electronic health record in the timeframe examined (Methods). Each panel represents a performance metric and each row within a panel represents a separate model. For each model, estimates are separated for the training and holdout (test) data. Although the test data are the primary focus, we also depict performance in the training data (de-emphasized with increased transparency) to illustrate the variability in performance in the holdout data, even when repeated 10-fold cross-validation was used in the training data. The “null model” is for comparison; it represents a simple baseline that always predicts the modal response on the outcome (in this case, PTSD-positive). Orange color and dashed lines correspond to SCID-PTSD models, blue and solid lines to EHR-PTSD models. NPV: negative predictive value; PPV: positive predictive value; ROC_AUC: area under the receiver operating characteristic curve. XGBoost corresponds to extreme gradient boosting machines (xgboost R package); Rand.Forest corresponds to random forests (ranger R package); Elas.Net corresponds to elastic net logistic regression (glmnet R package).

PTSD status in the EHR was discordant from the SCID-5 criterion in 358 (26.7%) of cases (17.8% false negatives and 8.9% false positives) and concordant in the rest (55.4% true positives and 17.9% true negatives). Figure 1 depicts performance metrics for the SCID-PTSD (orange, dashed lines) and the EHR-PTSD models (blue, solid lines).

For comparison, the “null” model at the bottom of each facet simply always predicted the mode (in this case, PTSD-positive). Examining performance in the test set (circles) revealed some clear patterns. Most notably, the EHR-PTSD models outperformed the SCID-PTSD models for nearly every type of model on nearly every performance metric; model performance was mediocre when predicting SCID-PTSD, driven by low specificity and NPV. The tree-based models (XGBoost and random forest) generally performed better than the elastic net models, although this difference was small on most metrics. Across SCID- and EHR-PTSD models, sensitivity was notably higher than specificity: all models were better at identifying the presence of their respective PTSD criterion than its absence. Considering high sensitivity scores, this suggests a relatively high proportion of false positives in these model predictions, a problem that was less severe in the EHR-PTSD models. In the sensitivity analyses, setting the EHR-PTSD criterion to 2 visits with a PTSD diagnostic code (rather than one), model performance was similar (or slightly better) compared to the primary EHR-PTSD models (refer to Multimedia Appendix 1 for specific performance metrics).

Information about model calibration and variable importance is detailed in Multimedia Appendix 1.

### Analysis of Model Performance Discrepancies

**Figure 2.**
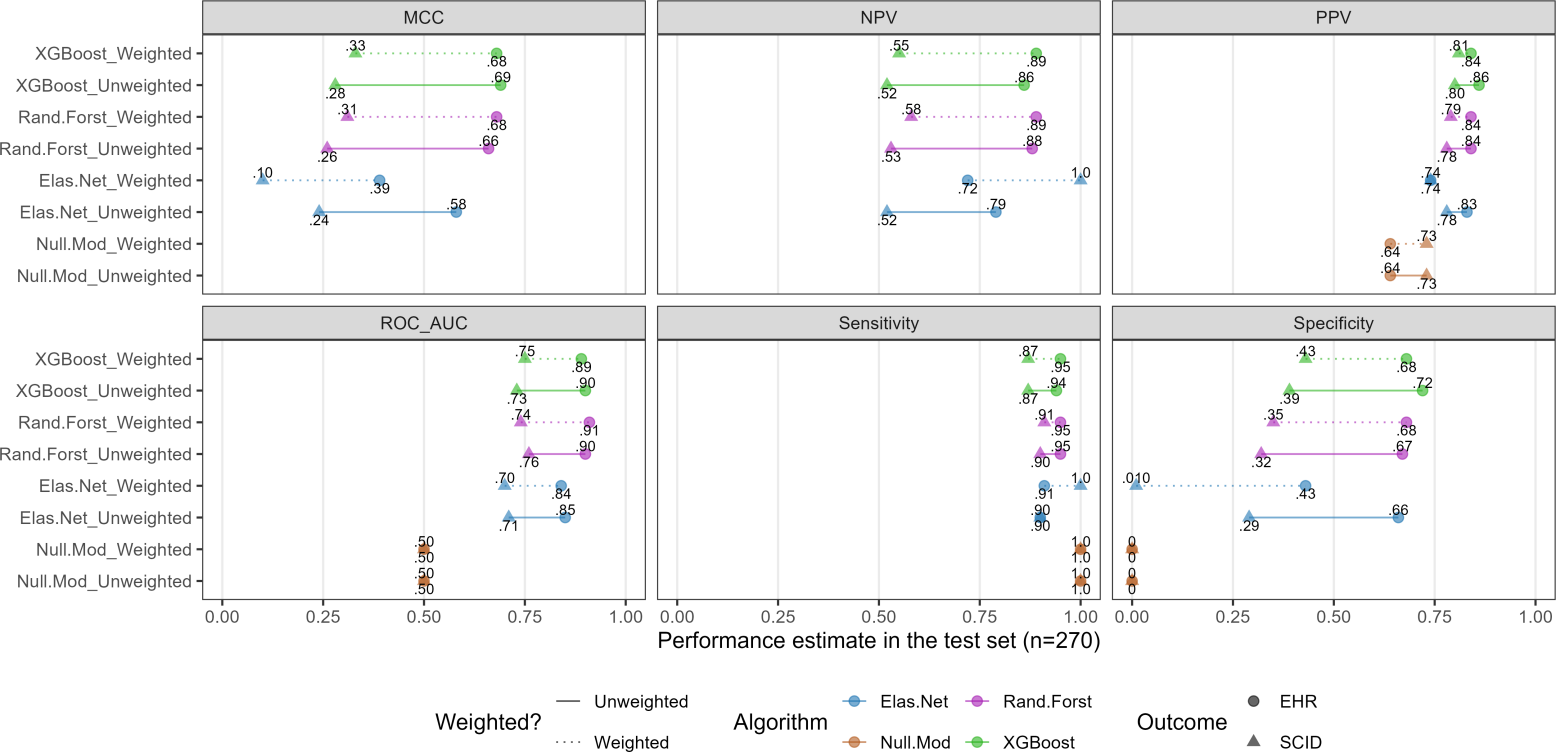
Performance metrics for SCID- and EHR-PTSD models weighted by data sparsity. SCID corresponds to models whose outcome was PTSD (yes/no) based on the SCID-5 semistructured diagnostic interview. EHR corresponds to models whose outcome was PTSD (yes/no) based on any history of diagnosis in the Veterans Affairs electronic health record in the timeframe examined (Methods). Only the estimates from the holdout (test) data are depicted here, for parsimony. NPV: negative predictive value; PPV: positive predictive value; ROC_AUC: area under the receiver operating characteristic curve. XGBoost corresponds to extreme gradient boosting machines (xgboost R package); Rand.Forest corresponds to random forests (ranger R package); Elas.Net corresponds to elastic net logistic regression (glmnet R package).

In a post hoc analysis examining the possibility that the better performance of the EHR-PTSD models relative to the SCID-PTSD models may relate to the number of visits observed in the EHR (ie, the density or sparsity of data for a given participant), we found that there were significant associations between EHR data sparsity (proportion of zeroes among each participant’s EHR variables) and PTSD status. Among participants with a PTSD diagnosis in the EHR, the proportion of zeroes was 0.65 (SD 0.16), significantly lower than the proportion among those without a history of PTSD diagnosis in the EHR, mean 0.89 (SD 0.14), Welch *t*_1101.9_=28.69, *P*<.001. The data sparsity difference was less pronounced among those with (mean 0.69 [SD 0.18]) versus without (mean 0.86 [SD 0.14]) a PTSD diagnosis on the SCID-5, Welch *t*_832.98_=17.69, *P*<.001. Multiple regression analysis showed EHR-PTSD (b=−0.18, SE 0.02, *P*<.001) was a stronger predictor of data sparsity than SCID-PTSD (b=−0.06, SE 0.01, *P*<.001) in a model that also included their interaction (b=−0.05, SE 0.02, *P*=.02). This model, which explained 39.3% of the variance in data sparsity, suggested that participants positive for either indicator of PTSD, but especially EHR-PTSD, had more populated EHR data, *F*_3,1339_=290.07, *P*<.001. When all models were rerun with importance weights (where participants with denser EHR data were given greater importance in model fitting), EHR-PTSD models were virtually unchanged, whereas the SCID-PTSD models performed slightly more poorly overall. This is depicted in Figure 2.

## Discussion

### Principal Findings

In this study of a large sample of U.S. veterans, multiple machine learning models using features from the EHR demonstrated limited accuracy in predicting PTSD diagnosed through structured interview conducted by well-trained assessors. Alternatively, the models performed very well when replacing the outcome with PTSD status in the EHR. This discrepancy held true regardless of algorithm type and regardless of whether the threshold for the dichotomous EHR-PTSD outcome was any (1+) PTSD diagnosis documented versus a minimum of 2. These predictive models of PTSD based solely on EHR data thus produced overoptimistic performance estimates relative to models predicting a rigorous PTSD criterion external to the EHR (ie, diagnostic interview). Past research has established that psychiatric diagnosis in the EHR is often discrepant from, not interchangeable with, an external criterion [11-13]. Our results suggest that this extends into multivariable models: outputs from clinical prediction models of these diagnostic indicators are likewise not interchangeable.

Secondary analyses probing possible reasons for the performance discrepancy between models with SCID-5 interview–based versus EHR-based PTSD criterion revealed that the presence of PTSD diagnosis (from either source) was associated with more populated EHR data (ie, fewer zeroes across the set of EHR variables for a given participant), likely because PTSD is associated with increased health care use [12,29]. Compared with SCID-5–based PTSD, EHR-based PTSD was especially strongly related to EHR data sparsity. We take this as evidence for a stronger circular relationship between health care use and EHR-based diagnosis, relative to interview-based diagnosis, and we believe this may be partially driving the observed performance differences between EHR-PTSD versus SCID-PTSD models.

In other words, the EHR-based PTSD outcome variable is implicitly capturing information about clinical service use that is also captured in the EHR-based predictors in the model, but is not captured in the external, interview-based SCID-5 measure of PTSD. Thus, our results are consistent with a circular process in which having PTSD increases the use of clinical services, and increased service use, in turn, increases the likelihood of being diagnosed with PTSD. But this circularity is only reflected in the EHR-PTSD models: frequently using VA services increases the chance that PTSD will be screened and diagnosed (regardless of actual PTSD status), and a PTSD diagnosis in the chart will be associated with increased visits due to the services made available to individuals with a diagnosis. This circularity is less reflected in the SCID-PTSD models because this more rigorous diagnosis should be only indirectly related to the frequency of VA service use (or ideally negatively correlated for individuals who recover as a function of receiving treatment). This would explain why weighting the models based on data sparsity barely affected the EHR-PTSD outcome, which already captured information about visit frequency, but substantially affected the SCID-PTSD outcome, which did not already capture such information. This circularity is also evident in the pattern of correlation among the study variables, as shown in the correlation matrix in Multimedia Appendix 1. There, we observed a pattern in which nearly all EHR variables were correlated with other EHR variables at least weakly, whereas they were correlated less strongly (if at all) with the non-EHR-based demographic variables.

Our findings complement those from past research highlighting several sources of bias in EHR data, including those related to missingness and nonrepresentativeness. For example, sicker patients generally use more services and thus have more data [9,16,30], and recent research on veterans’ health care use confirms that mental distress is among the conditions overrepresented in the VA EHR [31]. Our study also extends past work on diagnostic inaccuracies in the EHR [11-13] by highlighting problems that affect not only estimates of PTSD prevalence and its correlates but also results of multivariable predictive models, which may be prone to unrealistic performance estimates due to diagnostic inaccuracies, service use-related biases, or both.

Our results and the conclusions we draw from them should be tempered by some important considerations. First, our sample was one of convenience with an intentional overrepresentation of female veterans and veterans with PTSD. Although we are not certain about how oversampling for PTSD-positive cases in Project VALOR might have affected model performance, it is possible that oversampling for PTSD may have increased the predictive ability of the EHR-PTSD models by increasing the chance that correlates and comorbidities of EHR-based PTSD were present in the EHR data. It is thus unlikely to expect that either of our models would perform particularly well in randomly-sampled real-world data, but we do not believe that this oversampling strategy can account for the substantive finding in this study, that is, the robustness of the discrepancy we observed between EHR-based and SCID-based PTSD prediction models. However, we were not able to test this directly using sampling weights, because the sampling weights (and the data used to produce them) were not available to us. Second, we were unable to make definitive conclusions about how widespread a problem the overoptimistic performance estimates in the EHR-PTSD models are, because the VALOR study was not designed to specifically address this issue. The ideal study design would randomly sample users of VA health care services and then assess using clinical interview to draw definitive conclusions about the specific impacts on predictive performance of (1) the circular relation between PTSD status in the EHR and VA service use and (2) the discrepancy between EHR-PTSD status and SCID-PTSD status. Notwithstanding these considerations, the Project VALOR data also represent a relative study strength given that high proportions of PTSD-positive cases and female participants allow better estimates related to these variables.

### Conclusions

The current work sounds a cautious note regarding sole reliance on EHR data for clinical prediction models in mental health research. Using EHR-coded diagnoses for predictive models may exaggerate performance and thus limit the validity of models whose predictors and outcome share a common source (the EHR) that is subject to the effect of unobserved confounders. Linking prediction to rigorous diagnostic criteria is ideal and may be integral in some contexts. Though interviews are burdensome to collect, they reflect the nuanced reality of PTSD much more accurately than the EHR data alone. For the foreseeable future, it appears unlikely that a quick, digital proxy will be any replacement.

## Supplementary material

10.2196/63352 Multimedia Appendix 1 Supplemental tables and figures for EHR-based machine learning models for PTSD prediction.

## Abbreviations

AUC: area under the receiver operating characteristic curve
CV: cross-validation
ED/UC: emergency department or urgent care
EHR: electronic health record
*ICD-10*: *International Statistical Classification of Diseases and Related Health Problems 10th Revision*
*ICD-9*: *International Classification of Diseases, Ninth Revision*
MCC: Matthews correlation coefficient
NPV: negative predictive value
OEF: Operation Enduring Freedom
OIF: Operation Iraqi Freedom
OPMH: outpatient mental health
PPV: positive predictive value
PTSD: posttraumatic stress disorder
SCID-5: Structured Clinical Interview for *DSM-5* (*Diagnostic and Statistical Manual of Mental Disorders, Fifth Edition*)
VALOR: Veterans After-Discharge Longitudinal Registry
VHA: Veterans Health Administration
