# Supplementary material for: Misleading Results in Posttraumatic Stress Disorder Predictive Models Using Electronic Health Record Data: Algorithm Validation Study
Source: J Med Internet Res. 2025 Aug 27;27:e63352. doi: 10.2196/63352 (PMC12384688; doi:10.2196/63352)
Supplement: Multimedia Appendix 1 [file jmir-v27-e63352-s001.pdf]

Table of Contents

Table of Contents 1

Supplement Table S01. EHR variables used in the current study 2

Supplement Table S02. Model performance for EHR-PTSD models when setting the cutoff for the PTSD criterion to minimum 2 (rather than 1) PTSD diagnoses in the EHR 4

Supplement S03. Hyperparameter tuning scheme and final model values 6

  Elastic net logistic regression (glmnet) 6

  Random forest classification (ranger) 6

  Gradient boosting classification (xgboost) 6

Supplement Figures S04. Calibration plots for XGBoost models 7

  Calibration for original models, i.e., with uncalibrated probabilities 7

    Calibration plot for *uncalibrated* SCID-PTSD XGBoost model 7

    Calibration plot for *uncalibrated* EHR-PTSD XGBoost model 8

  Post-calibration information for XGBoost SCID-PTSD model 8

Supplement Figures S05. Variable importance plots 9

  SCID-PTSD models variable importance 9

  EHR-PTSD models variable importance 10

Supplement S06. Correlation Matrix 11

References 12

Supplement Table S01. EHR variables used in the current study

VARIABLE	MEAN	SD	MEDIAN	SKEW	KURTOSIS	RANGE	DESCRIPTION
**EDUC_PSYCH_DX_ATVISIT1**	0.30	1.52	0	13.89	261.98	35	Count of ED/UC visits where at least one diagnostic at the visit is a psychiatric disorder
**EDUC_PTSDDX_ATVISIT1**	0.15	0.85	0	13.90	272.19	20	Count of ED/UC visits where a PTSD diagnosis code assigned at the visit. (This was one of the two variables that was collapsed into a single binary variable that constituted the outcome variable in the EHR-PTSD models. In those models, it was not used as a predictor.)
**OPMH_ASSESS_EVAL1**	0.95	1.71	0	3.23	14.07	15	Count of OPMH visits coded as assessment/evaluation/intakes
**OPMH_EMCODEPRESENT1**	3.52	7.15	1	4.66	30.79	76	Count of OPMH visits with an Evaluation and Management service code
**OPMH_FAMILY_THERAPY1**	0.30	2.28	0	12.63	191.20	45	Count of OPMH visits coded as family therapy
**OPMH_GROUP_PSYCHOTHER1**	6.11	20.68	0	6.97	67.80	285	Count of OPMH visits coded as traditional group therapy
**OPMH_HEALTH_BEHAV_GROUP1**	0.14	1.82	0	23.63	653.95	55	Count of OPMH visits coded as health and behavior interventions for group or family
**OPMH_HEALTH_BEHAV_INDIVID1**	0.05	0.45	0	11.99	176.85	9	Count of OPMH visits coded as health and behavior interventions for individuals
**OPMH_INDIV_THERAPY_WITHMED1**	4.12	6.67	1	3.56	20.96	70	Count of OPMH visits coded as individual psychotherapy with evaluation/management services
**OPMH_INDIVIDPSYCHOTHERAPY1**	9.64	17.98	2	3.44	16.81	184	Count of OPMH visits coded as individual psychotherapy without evaluation/management services
**OPMH_MED_NO_THERAPY1**	2.97	5.72	0	3.81	21.49	57	Count of OPMH visits coded as office visit with prescriber without psychotherapy
**OPMH_MHPRIMARYCARE1**	1.51	5.92	0	8.21	95.13	104	Count of OPMH visits coded as occurring in Primary Care mental health
**OPMH_OCC_VOC_COMM1**	0.42	3.86	0	19.05	442.72	104	Count of OPMH visits coded as occupational/vocational/community services
**OPMH_OTHER_GROUP1**	1.24	6.88	0	8.51	88.78	107	Count of OPMH visits coded as group therapy other than traditional group therapy
**OPMH_OTHER_INDIV_COUNS1**	1.04	19.09	0	35.20	1267.73	691	Count of OPMH visits coded as other individual educational and counseling services (outside traditional psychotherapy CPT codes)
**OPMH_OTHER_TX1**	2.23	14.05	0	20.02	533.38	409	Count of OPMH visits coded as visit types not otherwise categorized in the OPMH data
**OPMH_PEER_SUPPORT1**	0.35	3.99	0	20.51	518.61	114	Count of OPMH visits coded as peer support visits
**OPMH_PHONE_OR_ONLINE1**	2.82	7.11	0	6.43	57.93	95	Count of OPMH visits phone or other non face to face encounters

VARIABLE	MEAN	SD	MEDIAN	SKEW	KURTOSIS	RANGE	DESCRIPTION
**OPMH_PSYCH_TESTING1**	0.20	0.81	0	6.84	62.93	11	Count of OPMH visits coded as psychological testing
**OPMH_PTSDDX_ATVISIT1**	18.35	34.69	6	5.37	49.99	502	Count of OPMH visits where a PTSD diagnosis code was assigned at the visit. (This was one of the two variables that was collapsed into a single binary variable that constituted the outcome variable in the EHR-PTSD models. In those models, it was not used as a predictor.)
**OPMH_SUDCLINIC1**	2.81	14.10	0	6.97	55.77	171	Count of OPMH visits that occurred in a SUD clinic of some type
**OPMH_TEAM_CONFERENCE1**	0.05	0.46	0	17.33	380.20	12	Count of OPMH visits coded as team conferences with patient present
**PHARM_ANTICONVULSANT1**	4.64	13.60	0	5.90	50.80	196	Count of pharmacy dispensations of anticonvulsant medications (besides clonazepam)
**PHARM_ANTIPSYCHOTIC1**	2.75	10.10	0	6.65	63.73	154	Count of pharmacy dispensations of antipsychotic medications
**PHARM_ANY_ANTIDEPRESSANT1**	15.54	20.66	8	2.32	8.44	187	Count of pharmacy dispensations of any antidepressant medication
**PHARM_BENZO1**	4.38	11.53	0	3.92	20.58	119	Count of pharmacy dispensations of tricyclics, MAOIs, SSRIs, and SNRIs; and mirtazapine, bupropion, and trazodone
**PHARM_OPIOID1**	4.26	12.71	0	4.98	32.62	158	Count of pharmacy dispensations of opioids
**PHARM_OTHER_SEDATIVES_HYPNOTICS1**	3.55	8.96	0	3.17	10.54	66	Count of pharmacy dispensations of non-benzo sedative/hypnotic medications
**PHARM_PRAZOSIN1**	2.15	8.02	0	6.23	50.31	102	Count of pharmacy dispensations of prazosin

Supplement Table S02. Model performance for EHR-PTSD models when setting the cutoff for the PTSD criterion to minimum 2 (rather than 1) PTSD diagnoses in the EHR

Below is the raw R output displaying the performance metrics for the EHR-PTSD models in which, instead of *any* (i.e., minimum 1) PTSD diagnosis in the chart as depicted in the primary EHR-PTSD models in the manuscript, a minimum of 2 diagnoses was used as the cutoff to classify a case as positive on the criterion. All combinations of performance metric, model/algorithm type, and train vs test/holdout set are shown. Performance changed only minimally compared to the EHR-PTSD models described in the manuscript, presumably because only a few participants (*n* = 51, 3.8% of the sample) had one and only one visit with a PTSD diagnosis rendered in the chart, and are thus the only participants whose data would cause a change in model performance. The other 812 participants (60.5%) with some history in the chart of a PTSD diagnosis had more than one visit with a PTSD diagnosis code.

“glmnet” refers to the elastic net models; “ranger” to the random forest models; and “xgboost” to the gradient boosting models.

```
   type  target     .metric      .estimate engine
   <chr> <chr>      <chr>            <dbl> <chr>
 1 test  ehr_2xptsd mcc              0.657 glmnet
 2 train ehr_2xptsd mcc              0.647 glmnet
 3 test  ehr_2xptsd bal_accuracy     0.814 glmnet
 4 train ehr_2xptsd bal_accuracy     0.814 glmnet
 5 test  ehr_2xptsd f_meas           0.873 glmnet
 6 train ehr_2xptsd f_meas           0.868 glmnet
 7 test  ehr_2xptsd sens             0.926 glmnet
 8 train ehr_2xptsd sens             0.904 glmnet
 9 test  ehr_2xptsd spec             0.701 glmnet
10 train ehr_2xptsd spec             0.724 glmnet
11 test  ehr_2xptsd ppv              0.825 glmnet
12 train ehr_2xptsd ppv              0.834 glmnet
13 test  ehr_2xptsd npv              0.862 glmnet
14 train ehr_2xptsd npv              0.832 glmnet
15 test  ehr_2xptsd roc_auc          0.883 glmnet
16 train ehr_2xptsd roc_auc          0.900 glmnet
17 test  ehr_2xptsd mcc              0.731 ranger
18 train ehr_2xptsd mcc              0.820 ranger
19 test  ehr_2xptsd bal_accuracy     0.846 ranger
20 train ehr_2xptsd bal_accuracy     0.893 ranger
21 test  ehr_2xptsd f_meas           0.900 ranger
22 train ehr_2xptsd f_meas           0.931 ranger
23 test  ehr_2xptsd sens             0.963 ranger
24 train ehr_2xptsd sens             0.985 ranger
25 test  ehr_2xptsd spec             0.729 ranger
26 train ehr_2xptsd spec             0.802 ranger
27 test  ehr_2xptsd ppv              0.844 ranger
28 train ehr_2xptsd ppv              0.884 ranger
29 test  ehr_2xptsd npv              0.929 ranger
30 train ehr_2xptsd npv              0.971 ranger
31 test  ehr_2xptsd roc_auc          0.914 ranger
32 train ehr_2xptsd roc_auc          0.988 ranger
33 test  ehr_2xptsd mcc              0.738 xgboost
34 train ehr_2xptsd mcc              0.765 xgboost
35 test  ehr_2xptsd bal_accuracy     0.852 xgboost
36 train ehr_2xptsd bal_accuracy     0.868 xgboost
37 test  ehr_2xptsd f_meas           0.902 xgboost
38 train ehr_2xptsd f_meas           0.912 xgboost
39 test  ehr_2xptsd sens             0.957 xgboost
```

```
40 train ehr_2xptsd sens            0.961 xgboost
41 test  ehr_2xptsd spec            0.748 xgboost
42 train ehr_2xptsd spec            0.774 xgboost
43 test  ehr_2xptsd ppv             0.852 xgboost
44 train ehr_2xptsd ppv             0.867 xgboost
45 test  ehr_2xptsd npv             0.920 xgboost
46 train ehr_2xptsd npv             0.929 xgboost
47 test  ehr_2xptsd roc_auc         0.912 xgboost
48 train ehr_2xptsd roc_auc         0.961 xgboost
```

Supplement S03. Hyperparameter tuning scheme and final model values

Multiple hyperparameters were tuned in R using k-fold cross-validation for 3 types of model: elastic net (logistic) regression using the glmnet package; random forest using the ranger package; and gradient boosted trees using the xgboost package. For each model type, the `tune_race_anova` function in the *finetune* R package was used for selecting the optimal hyperparameter set, using AUROC as the criterion for selection. The optimal hyperparameters for each tuned parameter are given for the main SCID-PTSD and EHR-PTSD models described in the manuscript.

Elastic net logistic regression (glmnet)

- Tuned 1) penalty (i.e., lambda) and 2) mixture (i.e., alpha)
- Grid was 250 rows of combinations of these parameters, automatically created using the `grid = 250` argument within the finetune::tune_race_anova_workflow function
- SCID models final hyperparameters: list(penalty = ~0.000678339830846581, mixture = ~0.116964863042813)
- EHR models final hyperparameters: list(penalty = ~0.139456342535512, mixture = ~0.135114604821196)

Random forest classification (ranger)

- Tuned 1) mtry (i.e., min_cols) and 2) min_n (i.e., min.node.size); otherwise, ranger package defaults were used
- Grid was every possible combination – using expand_grid() function – of mtry = c(3,4,5) and min_n = c(10,15,25,30)
- SCID models final hyperparameters: list(mtry = ~3, trees = ~2500, min_n = ~25)
- EHR models final hyperparameters: list(mtry = ~4, trees = ~2500, min_n = ~15)

Gradient boosting classification (xgboost)

- Tuned 1) mtry (i.e., colsample_bynode), trees (i.e., nrounds), min_n (i.e., min_child_weight), and learn_rate (i.e., eta); otherwise, xgboost package defaults were used
- Grid was 250 rows of combinations of these parameters, automatically created using the `grid = 250` argument within the finetune::tune_race_anova_workflow() function
- SCID models final hyperparameters: list(mtry = ~37, trees = ~171, min_n = ~3, learn_rate = ~0.003477232791913)
- EHR models final hyperparameters: list(mtry = ~11, trees = ~1321, min_n = ~6, learn_rate = ~0.00280237516178867)

Supplement Figures S04. Calibration plots for XGBoost models

XGBoost models evidenced relatively poor calibration for the SCID-PTSD model and good calibration for the EHR-PTSD model (ICI_{SCID} = .13; ICI_{EHR} = .02). Calibration plots based on logistic generalized additive models (GAMs) are shown in the figures below. The SCID-PTSD XGBoost model was well-calibrated after applying logistic GAMs to the original model probabilities ($ICI_{post\text{-}calibration}$ = .01).

Calibration for original models, i.e., with uncalibrated probabilities

The plots below were created in R using the ` rms::val.prob()` function. They depict multiple calibration-related performance metrics. The "Eavg" depicted on the plots is equivalent to the integrated calibration index named in the main paper [1], which is the mean absolute difference between observed and predicted probabilities. In other words, it is a measure of how much, on average, the predicted probabilities line up with the observed frequencies of PTSD.

Calibration plot for *uncalibrated* SCID-PTSD XGBoost model

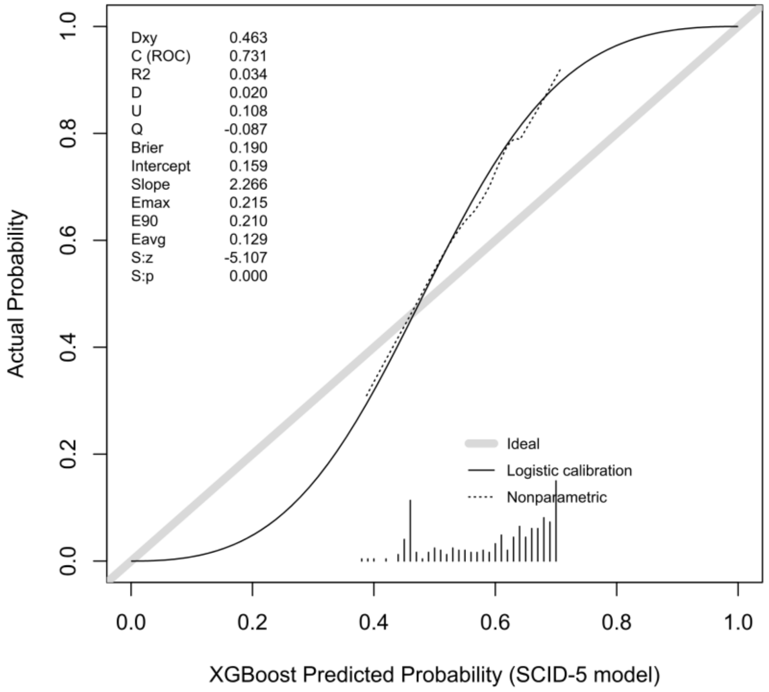

Calibration plot for *uncalibrated* EHR-PTSD XGBoost model

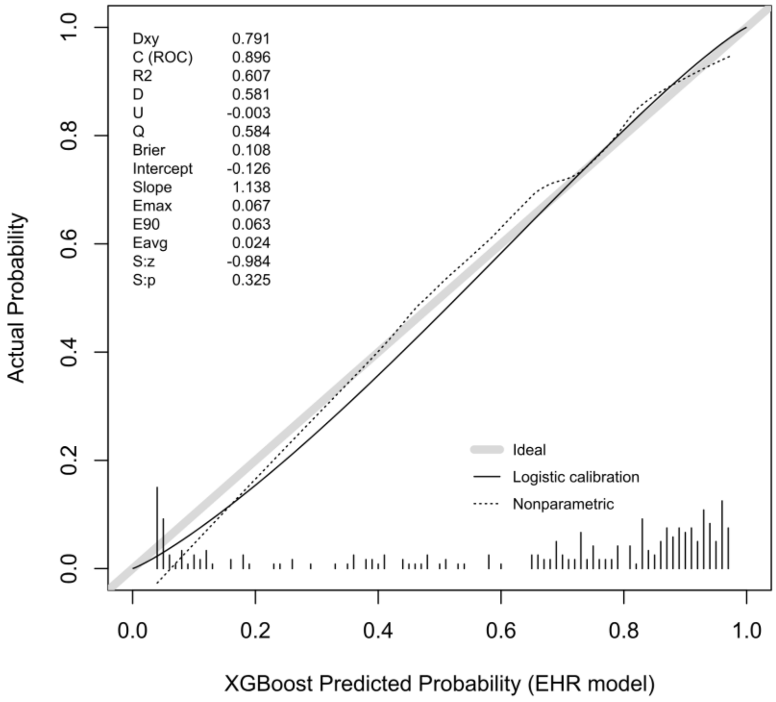

Post-calibration information for XGBoost SCID-PTSD model

The plot/information is only shown for calibrated SCID-PTSD (but not EHR-PTSD) model because probabilities from the EHR-PTSD XGBoost model were sufficiently calibrated in their original (i.e., untransformed) state. Post-calibration performance metrics are also shown after the plot.

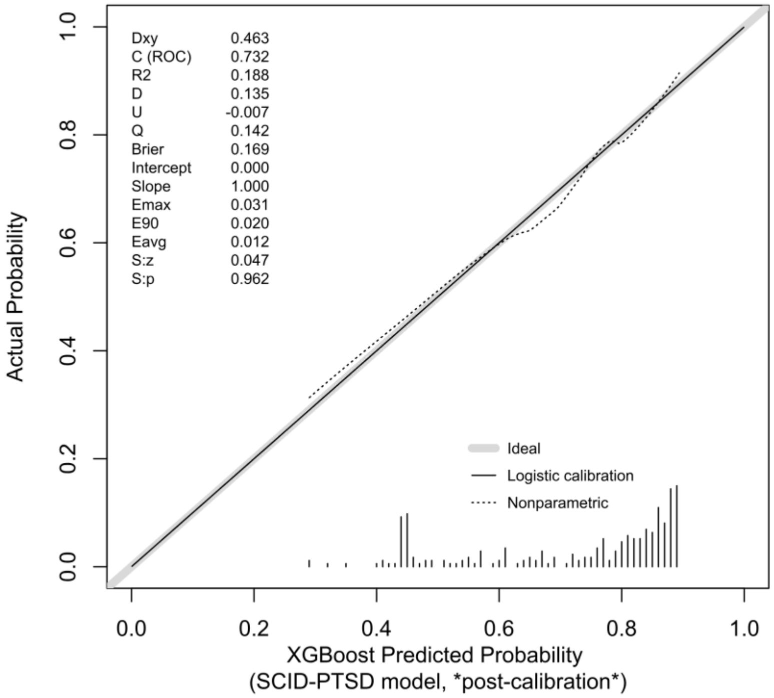

METRIC	ESTIMATE
**MCC**	0.294
**BAL_ACCURACY**	0.629
**F_MEAS**	0.837
**SENS**	0.883
**SPEC**	0.375
**PPV**	0.795
**NPV**	0.540

Supplement Figures S05. Variable importance plots

The 15 most important variables for each model are provided here for reference. Variable importance (on the x-axis) is calculated differently for each type of model and thus should not be directly compared across model types (e.g., elastic net with random forest). Although these may provide some initial evidence about what variables are most relevant for building predictive models of PTSD with data from the EHR, they should be considered preliminary at best, given that 1) inference was not our primary goal and 2) there are known issues with permutation and similar methods for variable importance in machine learning models [2]. These issues are beyond the scope of the current paper, but overall the variable importance methods used here should not be seen as reliable for inference about the relative impact of individual variables.

SCID-PTSD models variable importance

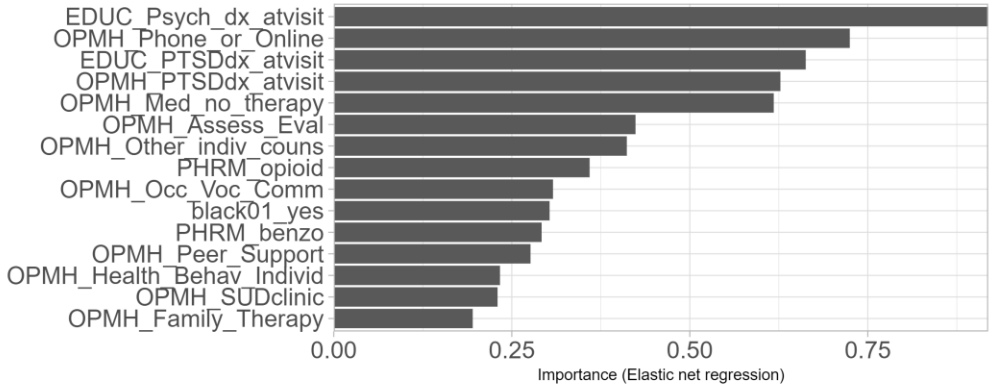

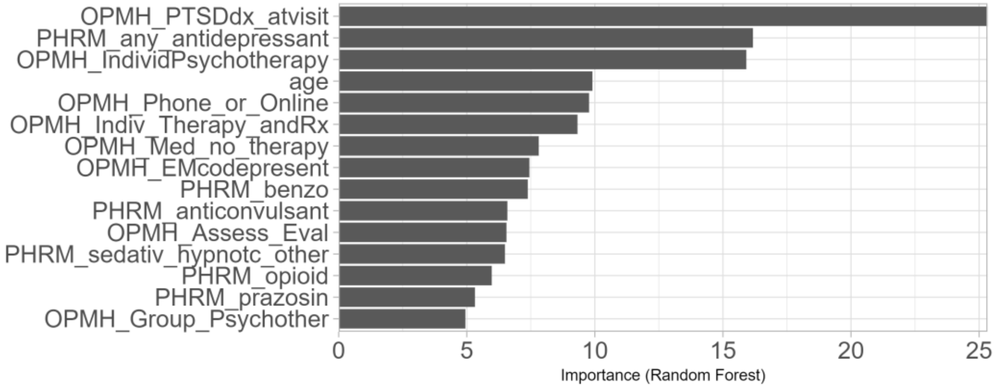

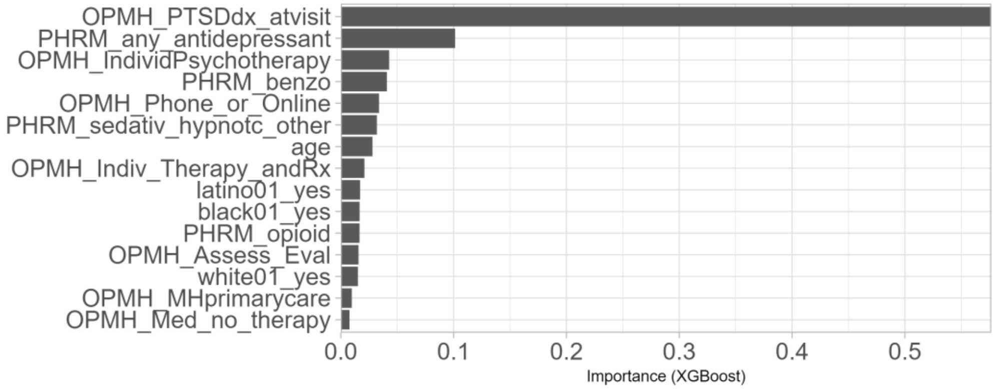

EHR-PTSD models variable importance

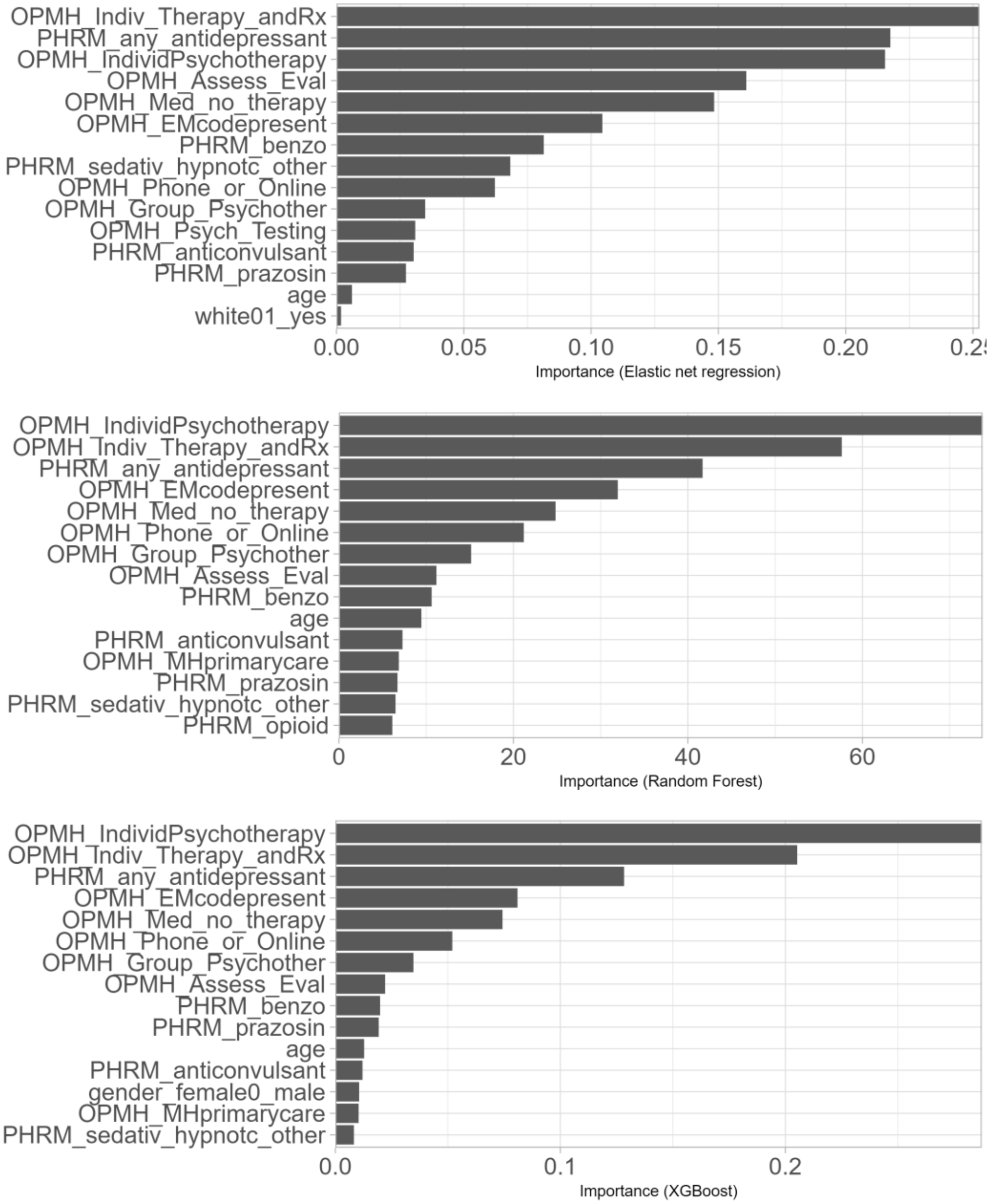

Supplement S06. Correlation Matrix

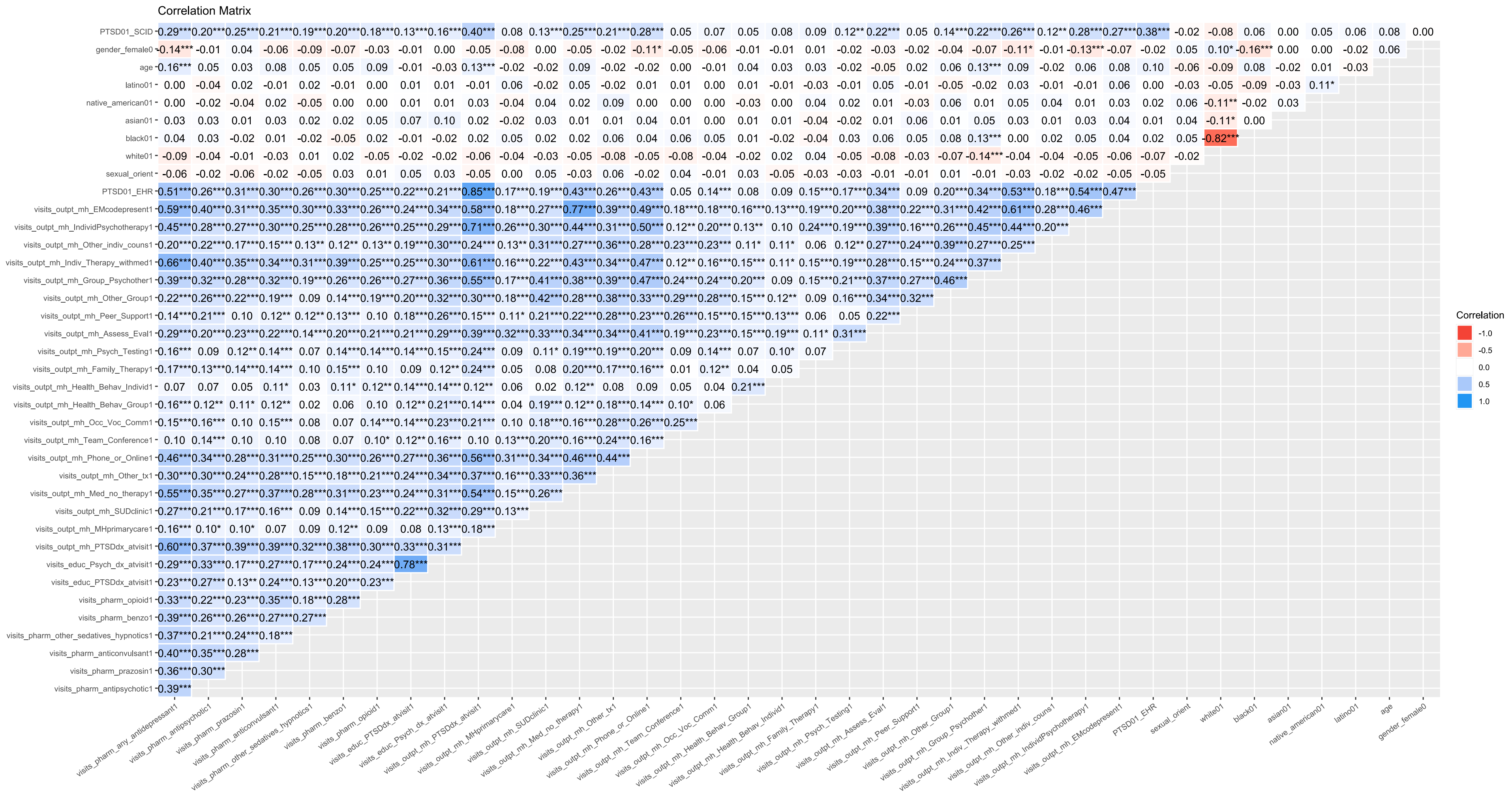

References

1. Austin PC, Steyerberg EW. The Integrated Calibration Index (ICI) and related metrics for quantifying the calibration of logistic regression models. *Stat Med*. 2019;38(21):4051-4065. doi:10.1002/sim.8281

2. Hooker G, Mentch L, Zhou S. Unrestricted permutation forces extrapolation: Variable importance requires at least one more model, or there is no free variable importance. *Stat Comput*. 2021;31(6):82. doi:10.1007/s11222-021-10057-z
